# Thoracoliths in Pleural Space Mimicking Lung Cancer: A Case Report

**DOI:** 10.7759/cureus.48522

**Published:** 2023-11-08

**Authors:** Ghulam Mustafa Aftab, Kristianna Fredenburg, Mindaugas Rackauskas, Pankit Patel, Hiren J Mehta

**Affiliations:** 1 Pulmonary Medicine, University of Florida College of Medicine, Gainesville, USA; 2 Pathology, University of Florida College of Medicine, Gainesville, USA; 3 Thoracic Surgery, University of Florida College of Medicine, Gainesville, USA; 4 Medicine/Pulmonary and Critical Care, University of Florida College of Medicine, Gainesville, USA

**Keywords:** video-assisted thoracic surgery, thoracic surgery, ct chest, lung cancer, thoracoliths, lung nodule

## Abstract

Pulmonary nodules often present a diagnostic challenge due to their diverse etiology, ranging from benign to malignant conditions. We discuss the diagnostic journey of a 71-year-old female patient with a history of kidney stones, who was incidentally found to have a pleural-based pulmonary nodule during a CT urogram. Subsequent imaging showed nodule growth, prompting further investigations, including a PET/CT scan and CT-guided biopsy, which yielded inconclusive results. A multidisciplinary approach recommended surgical resection, revealing three mobile calcified-like nodules within the pleural space, later identified as hyalinized nodules. The absence of malignancy was reassuring.

These benign, mobile pleural bodies, known as thoracoliths, are challenging to differentiate from pulmonary nodules. This case underscores the importance of considering rare benign entities in pulmonary nodule differentials and highlights the need for a multidisciplinary approach, surgical intervention, and open-mindedness in complex diagnostic scenarios.

## Introduction

Pulmonary nodules have long been considered a vexing challenge in clinical practice, embodying a complex diagnostic dilemma. Encountered frequently, these discrete lesions in the lung parenchyma can be harbingers of both benign and malignant conditions. Their evaluation necessitates a nuanced approach that aims to rule out malignancy while avoiding unnecessary invasive procedures. This case report delves into an instance of this diagnostic conundrum, involving a 71-year-old female patient with a history of kidney stones. Her initial presentation stemmed from the incidental discovery of a pulmonary nodule during a routine CT urogram in July 2022. The sequence of events over the next few months illustrates the critical need to consider rare benign entities, such as thoracoliths, within the differential diagnosis of pulmonary nodules that can closely mimic malignancy.

Pulmonary nodules are a common radiological finding, often detected incidentally during imaging studies for unrelated health concerns, as was the case with our patient. The challenge lies in differentiating between these nodules, which can encompass a broad spectrum of etiologies, ranging from benign granulomas, infections, and inflammatory processes to potentially life-threatening malignancies like lung cancer. Timely and accurate diagnosis is crucial as it dictates the subsequent clinical management and interventions.

The clinical significance of pulmonary nodules is underscored by the ever-evolving landscape of medical imaging technologies. High-resolution CT (HRCT) scans, PET/CT, and MRI have revolutionized the detection and characterization of these nodules, thereby enabling more precise assessment of size, shape, density, and metabolic activity. However, even with these advanced diagnostic tools at our disposal, the quest for an accurate diagnosis remains fraught with complexity as illustrated in our case discussed below.

## Case presentation

**Figure 1:**
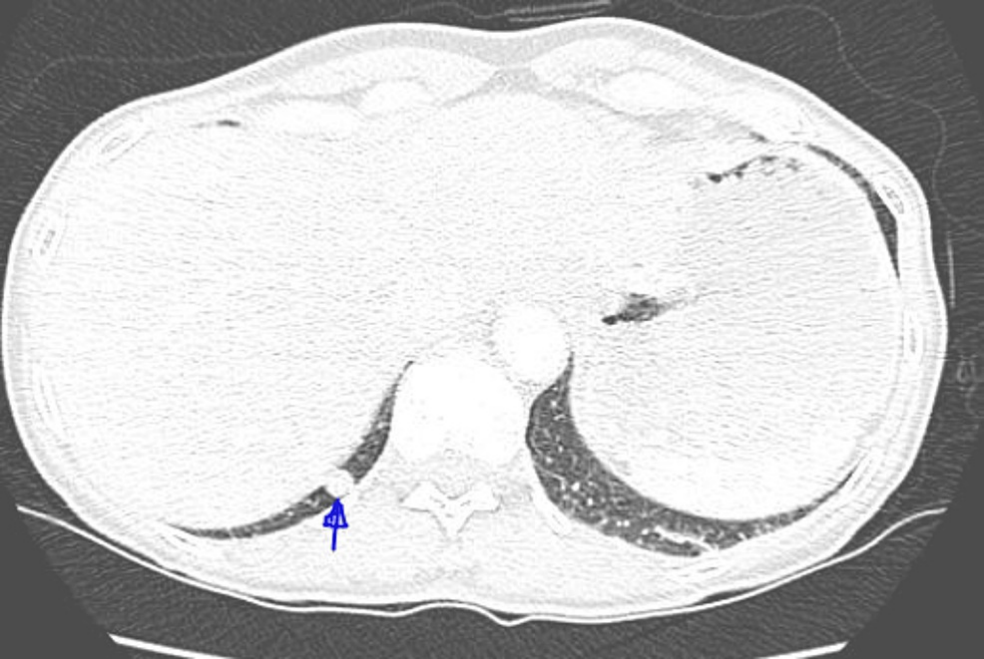
CT chest from August 2022 showing small RLL nodule CT: computed tomography; RLL: right lower lobe

**Figure 2:**
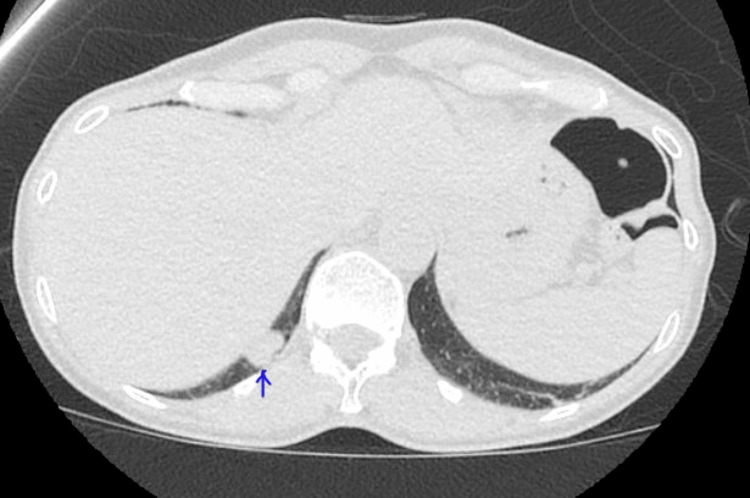
Repeat CT chest a year later showing interval enlargement of the RLL nodule CT: computed tomography; RLL: right lower lobe

Our patient initially underwent a CT urogram in July 2022 to assess her kidney stones, which revealed an incidental pulmonary nodule, setting in motion a series of investigations. Subsequent CT chest scans at three and six months from her original CT urogram revealed a concerning trend: the right lower lobe nodule was growing in size, increasing from 12 to 16 mm (Figures 1, 2). The nodules did not have any calcifications to suggest benign etiology and the rate of growth and potential volume doubling time raised concerns of malignancy. These prompted further investigations.

**Figure 3:**
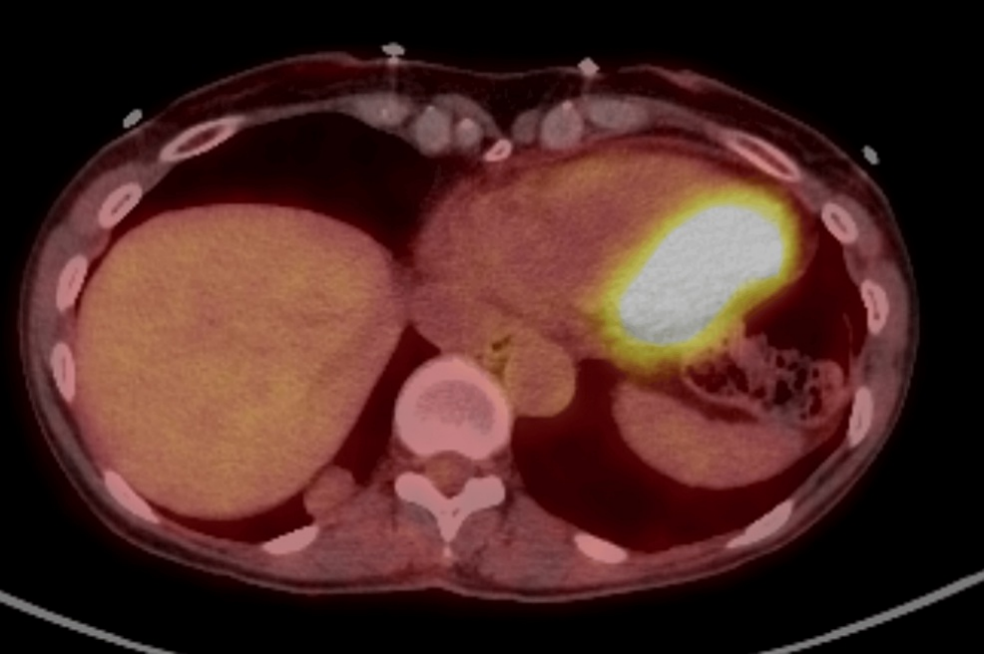
PET/CT scan showing the nodule is not FDG-avid FDG: fluorodeoxyglucose; PET/CT: positron emission tomography/computed tomography

A year later, a PET/CT scan was performed to evaluate the metabolic activity of the nodule (Figure 3). It indicated that the nodule was not avid for fluorodeoxyglucose (FDG). A CT-guided biopsy was attempted at that point but provided non-diagnostic results, consisting of bland mesothelial cells.

The complexity of the case necessitated a multidisciplinary approach. In light of the increase in nodule size and the inconclusive biopsy findings, a tumor board recommended surgical resection as the most appropriate course of action. This decision led to a robotic surgical procedure using the Da Vinci Xi system in May 2023.

**Figure 4:**
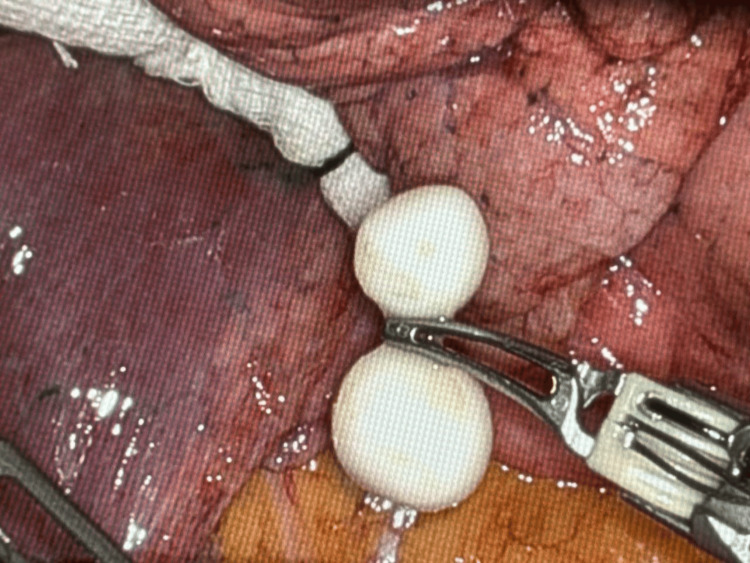
Intraoperative image showing free-floating nodules in the pleural cavity

**Figure 5:**
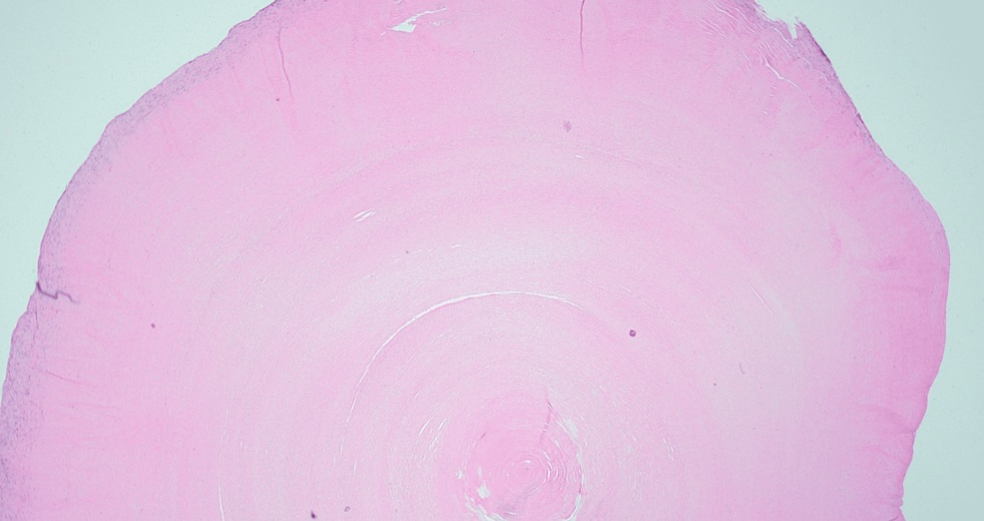
Representative section showing a well-circumscribed nodule with densely eosinophilic collagenous material best described as hyalinized (2X)

Intraoperatively, three free-moving round nodules at the level of the right lower lobe in the pleural space (Figure 4) were found and were removed. No other nodules were identified. No lung parenchyma was resected. The nodules that appeared to be intraparenchymal on imaging were actually pleural nodules with lung parenchyma, completely normal. Pathology showed hyalinized nodules (Figure 5).

**Figure 6:**
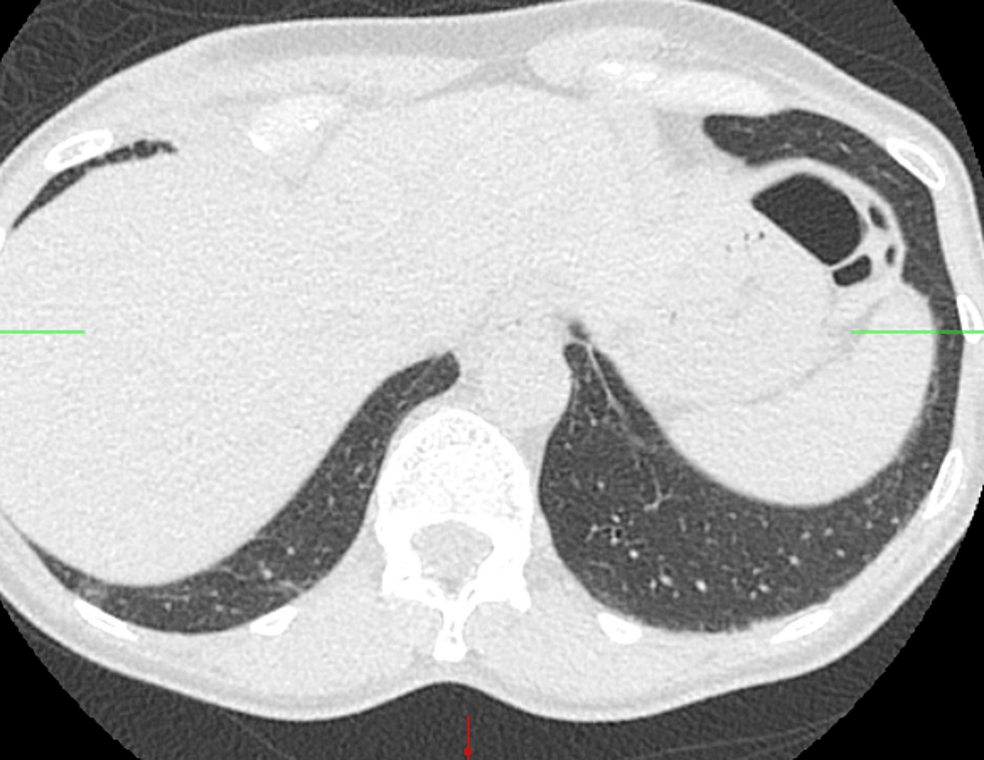
CT image 3 months post-surgery showing no residual nodules CT: computed tomography

The patient was followed up three months later and underwent a repeat CT chest, which showed no residual disease (Figure 6); she is currently doing well and has fully recovered from surgery.

## Discussion

Pulmonary nodules represent a diagnostic challenge due to the myriad possible underlying causes. They can range from benign lesions to malignant tumors, and distinguishing between them is paramount for appropriate clinical management. In this case, the pivotal discovery was not made until the surgical intervention. Intraoperatively, the surgical team identified three intriguing entities within the pleural space at the level of the right lower lobe: free-moving calcified-like nodules (Figure 4). These nodules were meticulously removed and subsequently subjected to pathological examination.

Pathological analysis of the resected nodules revealed a well-defined structure characterized by densely eosinophilic collagenous material (Figure 5). These nodules exhibited characteristics consistent with hyalinization. Notably, there was no indication of malignancy. Benign, mobile loose bodies in the pleural cavity are known as thoracoliths. These may or may not be calcified and are difficult to distinguish from pulmonary nodules [1]. Histology of these mobile bodies usually consists of a fatty core with or without necrosis. In some cases, as in our patient, it may consist of hyalinized collagen without a fatty core [2].

Various theories have been proposed regarding the origin of these thoracoliths, including degenerated pleural lipomas [3] (a rare entity themselves); past history of tuberculosis; phagocytosis of dust particles by macrophages [4]; or migration of pleural or pericardial fat into the pleural space [5]. However, none of these theories have been validated. The relationship between pericardial fat and thoracolithiasis is supported by the predominantly left-sided occurrence (70%). The differential diagnosis includes fibrin bodies, which would require a history of pleural effusion, hemorrhage or pneumothorax [6], or a foreign body granuloma [7]. A case of gallstone migration into the pleural space following laparoscopic cholecystectomy has also been described [8]. Thoracolith should be considered in the differential diagnosis of a peripheral lung nodule.

Our case is unique in that it involved right-sided thoracoliths mimicking pulmonary parenchymal nodules.

## Conclusions

This case report highlights the intricate and sometimes unpredictable nature of pulmonary nodules. The initial discovery of a nodule during a routine imaging study led to a series of investigations, each presenting unique challenges and raising concerns about malignancy. The ultimate identification of hyalinized nodules, or thoracoliths, within the pleural cavity served as a valuable lesson in considering rare benign entities when confronted with pulmonary nodules that may mimic lung cancer. The case also underscores the importance of a multidisciplinary approach, as well as the necessity for close collaboration between clinicians, radiologists, and pathologists when assessing pulmonary nodules. Furthermore, it lays stress on the critical role of surgical intervention in reaching a definitive diagnosis in complex cases where noninvasive methods prove inconclusive. As healthcare providers, we must remain vigilant and open to the possibility of unexpected findings, even when encountering radiological and clinical evidence that may suggest a more ominous diagnosis.
